# An FTA card-based CRISPR/Cas12a platform with isothermal amplification for portable tuberculosis diagnosis

**DOI:** 10.3389/fmicb.2026.1958414

**Published:** 2026-09-29

**Authors:** Jiayin Xing, Xiangxiang Zhao, Gaojie Wang, Shilong Ren, Lei Chen, Wenbin Diao, Meihua Qu, Zhimin Chang, Xiaoqian Wang, Shihao Zhao, Yong Zhang, Ningning Song

**Affiliations:** 1 Weifang Key Laboratory of Respiratory Tract Pathogens and Drug Therapy, School of Life Science and Technology, Shandong Second Medical University, Weifang, China; 2 Weifang No.2 People's Hospital, Weifang, Shandong, China

**Keywords:** CRISPR diagnostics technology, FTA card, isothermal amplification, point-of-care testing, tuberculosis

## Abstract

*Mycobacterium tuberculosis* remains a major global public health threat, imposing significant socioeconomic burdens. Current molecular diagnostics rely heavily on nucleic acid extraction and specialized equipment, which limits their use in resource-limited settings. We developed a rapid, sensitive platform suitable for point-of-care detection by integrating FTA cards with ERA/RT-ERA amplification and CRISPR/Cas12a, enabling direct analysis of samples without conventional nucleic acid purification. Leveraging FTA cards for efficient nucleic acid release and stable preservation, we established assays targeting IS6110, esxB, gyrB, and the RNA target ASdes. Integration of FTA-based sample processing, ERA/RT-ERA amplification, and Cas12a detection enables fast and reliable identification of nucleic acid markers. The detection limits were 12.5 pM for IS6110, 6.25 pM for esxB, 25 pM for gyrB, and 12.5 pM for ASdes. Our results showed strong concordance between endpoint fluorescence and qPCR results, demonstrating robust quantitative performance for both DNA and RNA targets. In clinical serum samples, the fluorescence readouts and lateral flow strip outputs showed strong concordance with quantitative PCR results across all four targets. Collectively, the FTA-ERA-CRISPR/Cas12a platform serves as a practical and offers a reliable approach for rapid tuberculosis diagnosis at the point of need. This integrated multiple distinct targets strategy provides a versatile framework for portable molecular diagnostics and holds promise for improving TB case detection in resource-limited settings.

## 1 Introduction

Tuberculosis (TB), caused by *Mycobacterium tuberculosis* (Mtb), remains one of the most burdensome infectious diseases worldwide (Kyu et al., 2024; GBD 2021 Tuberculosis Collaborators, 2024). According to the World Health Organization's Global Tuberculosis Report 2025, an estimated 10.7 million new TB cases occurred globally in 2024, with approximately 1.23 million deaths attributed to the disease (World Health Organization, 2025). Rapid and sensitive diagnostic methods are therefore essential for early case detection and timely initiation. Conventional sputum smear microscopy is simple and inexpensive, but its sensitivity is limited. False-negative results are particularly common in patients with low bacillary loads, including those co-infected with HIV, children, and individuals with extrapulmonary TB (Matee et al., 2008; Cattamanchi et al., 2009; Asare et al., 2024). Although *Mycobacteria* Growth Indicator Tube (MGIT) culture offers improved diagnostic performance, the prolonged turnaround time reduces its utility for early clinical decision-making (Musisi et al., 2024). Nucleic acid amplification tests based on PCR, such as Xpert^®^ MTB/RIF, provide higher sensitivity and enable simultaneous detection of TB and rifampicin resistance. However, they rely on dedicated instruments and single-use cartridges, which restrict their deployment in resource-limited settings (Bell et al., 2025). There is therefore a clear need for a TB molecular diagnostic approach that combines high sensitivity, rapid turnaround, low cost, and suitability for point-of-care testing.

In recent years, Clustered Regularly Interspaced Short Palindromic Repeats (CRISPR) and its associated Cas proteins have emerged as powerful tools in both genome engineering and nucleic acid diagnostics (Sahel et al., 2024; Li et al., 2023). CRISPR/Cas12a-based diagnosis is built on a ribonucleoprotein complex formed by Cas12a and its guide RNA (crRNA), which recognizes target dsDNA in a protospacer-adjacent motif (PAM)-dependent manner. Upon target binding, Cas12a is activated to exert collateral nuclease activity, cleaving reporter probes to generate a readily detectable fluorescent signal (Xiao et al., 2023). To improve practicality for on-site testing, Cas12a is often coupled with isothermal amplification strategies, such as Enzymatic Recombinase Amplification (ERA), enabling highly sensitive detection without thermal cycling (Shao et al., 2023; Xu et al., 2023; Feng et al., 2021). ERA uses enzyme-engineered recombinases to rapidly amplify trace amounts of DNA at a constant temperature (approximately 37–42 °C) within 15–20 min, reducing non-specific amplification and improving reaction fidelity (Gan et al., 2024). Gan et al. combined ERA with CRISPR/Cas12a for Mtb detection, achieving a limit of detection of 9 copies/μL with fluorescence readout and 90 copies/μL with lateral flow strips in approximately 50 min (Zhou et al., 2024). Beyond ERA, recombinase polymerase amplification (RPA)-CRISPR/Cas12a platforms have been widely applied for pathogen detection, including Mycoplasma pneumoniae and Mycoplasma hominis, integrating isothermal amplification with Cas12a-mediated fluorescence and lateral flow readouts and showing good agreement with qPCR (Zeng et al., 2025; Li et al., 2023). Compared with RPA, ERA offers a similarly rapid isothermal strategy with simplified reaction requirements and compatibility with streamlined workflows, making it suitable for portable diagnostic platforms.

In point-of-care molecular diagnostic systems, the choice of sample pretreatment strategy largely determines the overall workflow complexity and its feasibility in resource-limited settings (Chao et al., 2025). Flinders Technology Associates (FTA) cards are composed of a cellulose matrix impregnated with chaotropic agents that inactivate pathogens, lyse cells, and immobilize nucleic acids, preserving sample integrity and biosafety while enabling long-term nucleic acid stability at room temperature (Taesuji et al., 2024; Cardona-Ospina et al., 2019). Nucleic acid release from FTA cards is straightforward and rapid, requiring minimal equipment. By replacing conventional nucleic acid purification procedures, such as silica column- or magnetic bead-based extraction, FTA card-mediated sample processing simplifies the pretreatment workflow and makes this approach particularly suitable for field-based molecular testing in remote or resource-constrained environments (Fonseca et al., 2024). Beyond simplified sample preparation, visual readout is another critical element for instrument-free diagnostics. Lateral flow assays (LFAs) are widely used as output formats for portable detection owing to their low cost, ease of operation, and direct visual interpretability; in such systems, isothermally amplified targets activate Cas12a trans-cleavage, cleaving dual-labeled reporter probes to generate a visible strip signal (Kakkar et al., 2024; Safenkova et al., 2025).

This study established a molecular diagnostic platform integrating rapid FTA card processing, ERA/RT-ERA isothermal amplification, and CRISPR/Cas12a detection for multiple distinct targets identification of Mtb nucleic acids. Here, the term ‘simplified sample processing' refers to the replacement of conventional nucleic acid purification with FTA card-mediated nucleic acid preservation and release; this does not represent direct detection from untreated clinical specimens, but rather a streamlined workflow that reduces operational complexity before amplification. ERA or RT-ERA is performed at a constant temperature (37–42 °C), followed by CRISPR/Cas12a trans-cleavage for specific target detection, with signals read in real time via fluorescence or visualized on lateral flow strips. The four complementary Mtb-associated biomarkers were selected based on their distinct diagnostic characteristics. IS6110, a multicopy insertion sequence varying from 0 to 25 copies per genome in the *M. tuberculosis* complex, was chosen as the primary DNA target for its analytical sensitivity (Jagielski et al., 2016). esxB, encoding CFP-10 of the ESX-1 secretion system and highly conserved across the Mtb complex, and gyrB, encoding the DNA gyrase B subunit and widely used for mycobacterial identification, were incorporated as conserved single-copy genomic targets providing redundancy independent of IS6110 copy-number variation (Guo et al., 2022). ASdes was included as an RNA-derived target to explore the feasibility of detecting transcription-associated Mtb signals in blood, given that Mtb-derived small RNAs, including ASdes, have been detected in plasma from patients with active tuberculosis (Landry and Ferguson, 2000); however, whether circulating ASdes reflects bacterial activity or treatment response requires further investigation. Each target was first assessed by PCR or RT-PCR amplification combined with the CRISPR/Cas12a fluorescence assay for performance optimization, and the system was then preliminarily evaluated using clinical serum samples, comparing CRISPR/Cas12a fluorescence results with qPCR (Xue et al., 2021; Chang et al., 2024; Huang et al., 2022).

Compared with recent ERA-CRISPR/Cas12a platforms focused solely on the multicopy element IS6110 with fluorescence readout, the present study advances the field primarily through the integration of complementary components rather than any single novel element: a multiple distinct targets panel spanning DNA and RNA markers of varying copy number, a dual-mode readout combining quantitative fluorescence with instrument-free lateral flow, and an FTA card-based workflow that avoids conventional nucleic acid purification. Collectively, these integrated features establish a versatile CRISPR/Cas12a platform for TB molecular diagnostics suitable for point-of-care applications.

## 2 Materials and methods

### 2.1 Reagents

The RT-based nucleic acid amplification kit (ERA method, Cat. No. KS102) and CRISPR lateral flow strips for visual detection (Cat. No. TS104) were obtained from Suzhou Xinda Gene Technology Co., Ltd. (Suzhou, China). All PCR primers and DNA templates used for in vitro transcription of crRNAs were synthesized by General Biol Co., Ltd. (Anhui, China). qPCR amplification was performed using the HiScript II One Step qRT-PCR SYBR Green Kit (Vazyme, Nanjing, China; Cat. No. Q221-01). cfRNA from serum or plasma samples was isolated with the miRcute Serum/Plasma miRNA Isolation Kit (Tiangen Biotech, Beijing, China; Cat. No. DP503). Circulating DNA (cfDNA) was extracted using the Blood/Plasma Cell-free DNA Isolation Kit (Tiangen Biotech, Beijing, China; Cat. No. DP339), according to the manufacturers' protocols. crRNAs were generated by *in vitro* transcription of the corresponding DNA templates using the HiScribe T7 High Yield RNA Synthesis Kit (New England Biolabs, USA). The transcription products were purified with the RNA Clean & Concentrator Kit (Zymo Research, USA) and subsequently used in Cas12a-based assays. All Cas12a reactions were carried out in CutSmart Buffer (New England Biolabs, USA; Cat. No. B6004S).

### 2.2 Clinical samples

A total of 31 clinical serum samples were included in this study, comprising 8 culture-positive cases and 23 cases that were positive by molecular testing. Of the 31 clinical serum samples, 20 were used for the FTA-ERA/RT-ERA-CRISPR/Cas12a integrated workflow validation, 10 were selected for CRISPR/Cas12a fluorescence vs. qPCR method comparison, and nuclease-free water controls (NTC) were included in all assays. Following collection, blood samples were centrifuged at 3,000 × g for 10 min at 4 °C to separate the serum. The resulting serum was aliquoted and stored at −80 °C until further analysis.

### 2.3 Expression and purification of Cas12a protein

*Escherichia coli* BL21 (DE3) competent cells harboring the pET28TEV-LbCas12a expression plasmid were inoculated into LB medium supplemented with the appropriate antibiotic and cultured overnight at 37 °C with shaking at 150 rpm. Protein expression was induced by the addition of isopropyl β-D-1-thiogalactopyranoside (IPTG) to a final concentration of 100 μM, after which the incubation temperature was lowered to 16 °C and the culture was continued for 12 h. Following induction, cells were harvested by centrifugation at 4,000 × g for 15 min and collected for subsequent protein purification. The harvested cell pellet was resuspended in lysis buffer composed of 50 mM Tris-HCl (pH 7.4), 200 mM NaCl, 2 mM dithiothreitol (DTT), and 5% glycerol. Immediately prior to lysis, an EDTA-free protease inhibitor cocktail (Sigma-Aldrich, Roche Diagnostics) was added to the suspension. Cells were disrupted by sonication on ice (3 s on / 3 s off, total 10 min), and the lysate was clarified by centrifugation. The clarified lysate was loaded onto a 1 mL HisTrap FF nickel-affinity column (GE Healthcare) equilibrated with binding buffer (50 mM Tris-HCl pH 7.4, 200 mM NaCl, 2 mM DTT, 5% glycerol, 30 mM imidazole). Weakly bound proteins were removed by washing with the same buffer containing 30 mM imidazole. The target protein was eluted with a linear imidazole gradient (30–500 mM imidazole) over 20 column volumes. Fractions containing the protein of interest were identified by SDS-PAGE, pooled, and desalted by dialysis against buffer A (50 mM Tris-HCl pH 7.4, 50 mM NaCl, 2 mM DTT). The desalted sample was applied to a HiTrap Q HP anion-exchange column (GE Healthcare) equilibrated with buffer A, and bound proteins were eluted with a linear 0.05–1.0 M NaCl gradient. The eluted target peaks were collected, pooled, and concentrated for further use. The concentrated protein was further purified by size-exclusion chromatography using a HiLoad 16/600 Superdex 200 pg column on an ÄKTA Explorer 100 FPLC system (GE Healthcare), equilibrated with 20 mM Tris-HCl pH 7.5, 1 M NaCl. The fractions corresponding to the target peak were analyzed by reducing SDS-PAGE to assess protein purity. Briefly, purified protein samples were mixed with SDS-PAGE loading buffer containing reducing agents, heated, and separated on SDS-polyacrylamide gels. Protein bands were visualized by Coomassie Brilliant Blue staining, and the final purity was estimated to be >95%. The purified LbCas12a was then concentrated, transferred into a storage buffer consisting of 20 mM Tris-HCl (pH 7.5), 1 M NaCl, and 50% glycerol, and stored at −80 °C.

### 2.4 Primer, crRNA, and reporter probe preparation

Specific PCR primers for standard template preparation were designed for the four target loci examined in this study (IS6110, gyrB, esxB, and ASdes), including IS6110-dsDNA-F and IS6110-dsDNA-R; gyrB-dsDNA-F and gyrB-dsDNA-R; esxB-dsDNA-F and esxB-dsDNA-R; and ASdes-T-F and ASdes-T-R (Supplementary Table S1). To design crRNAs corresponding to these targets, crRNA sequences were generated using an online CRISPR RNA design tool (https://www.zlab.bio/) in combination with sequence information retrieved from the NCBI database. In addition, two types of reporter probes were designed, namely Reporter 1 (F-Q) for fluorescence-based detection and Reporter 2 (F-B) for lateral flow strip-based readout. Standard DNA templates were generated by PCR amplification using target-specific primers. The amplicons were verified by agarose gel electrophoresis, purified, and quantified. A series of standard templates ranging from 51.2 nM to 0.10 nM was then prepared by two-fold serial dilution, with a no-template control included in parallel.

crRNAs were prepared using a PCR-based strategy. Briefly, ssDNA templates encoding the target crRNA sequences were amplified with an upstream primer containing a T7 promoter, followed by *in vitro* transcription. Transcription reactions were carried out using the HiScribe T7 High Yield RNA Synthesis Kit (NEB) in a total volume of 30 μL, consisting of 10 μL NTP buffer mix, 2 μL T7 RNA polymerase, 3 μL PCR template, and 15 μL RNase-free water, and incubated at 37 °C for 16 h. The resulting crRNAs were purified with the RNA Clean & Concentrator Kit (Zymo Research) and used as key components in subsequent CRISPR/Cas12a assays. To accommodate different detection formats, two types of short ssDNA reporter probes with identical sequences but distinct terminal labels were designed. For fluorescence detection, F-Q probes were labeled with 5′-FAM and 3′-BHQ1, whereas for lateral flow-based detection, F-B probes were labeled with 5′-FAM and 3′-biotin. Upon target-dependent activation of Cas12a, both probes undergo collateral cleavage, resulting in fluorescence signal enhancement or visible readout on lateral flow strips, respectively. The sequences and applications of crRNAs and reporter probes are summarized in Table 1.

**Table 1:** Sequences of crRNA and probes used in the detection system and primers used for template amplification in qPCR assays.

Name	Sequence (5^′^ → 3^′^)
crRNA-IS6110	UAAUUUCUACUUGUAGAUGAUCGAGCCAGAACAUAU
crRNA-gyrB	UAAUUUCUACUUGUAGAUACGUGUUGGCGGCGACAUG
crRNA-esxB	UAAUUUCUACUUGUAGAUACUCGCCUAGAGGCCGCUG
crRNA-ASdes	UAAUUUCUACUUGUAGAUAGCCUGGGCUGAAGCCCCG
Reporter 1 (F-Q)	FAM-TTATT-BHQ1
Reporter 2 (F-B)	FAM-TTATT-Biotin
IS6110-F	GGTCGGAAGCTCCTATGACAATGCACTAGCC
IS6110-R	TTGAGCGTAGTAGGCAGCCTCGAGTTCGAC
esxB-F	TAAAGAGAGAAAGTAGTCCAGCATGGCAGAG
esxB-R	TCTCGTCGAGTTCCTGCTTCTGCTTATTGG
gyrB-F	CTGTTGTTGACGTTGTTGTTCCGGTTCATGC
gyrB-R	CTGAATGCCGTCTTCCTTGTTGATCTTCTTC
ASdes-F	TGAGGCTCTCCGCGAAATAG
ASdes-R	AGAAACTTCGCCTCGAGGTA
ERA-IS6110-F	GGTCGGAAGCTCCTATGACAATGCACTAGCC

### 2.5 Establishment and optimization of the CRISPR/Cas12a fluorescence assay

A CRISPR/Cas12a-based fluorescence detection platform was established and optimized for the rapid detection of four *tuberculosis*-related targets (IS6110, esxB, gyrB, and ASdes). To ensure robustness and comparability across targets, all assays were performed using an identical reaction composition and a unified optimization strategy, enabling reliable multiple distinct targets detection. Each reaction was carried out in a total volume of 30 μL, consisting of 0.75 μL Cas12a (10 μM), 0.75 μL crRNA (10 μM), 3 μL 10 × CutSmart Buffer, 0.6 μL F-Q reporter (50 μM), and 3 μL dsDNA template, with nuclease-free water added to reach the final volume. Under these conditions, Cas12a and crRNA were each added at a final concentration of 250 nM, and the F-Q reporter was present at 1 μM. All reactions were incubated at 37 °C and monitored in real time for fluorescence signals. Reaction mixtures were loaded into black 384-well plates and measured using a SpectraMax i3x multimode microplate reader. Fluorescence excitation and emission wavelengths were set to 485 nm and 520 nm, respectively. Signals were recorded every 1.5 min over a 60-min period. These conditions were selected to evaluate the relationship between target template concentration and fluorescence output, and to define the dynamic range and analytical sensitivity of the assay.

To optimize the reaction system and improve analytical sensitivity, key parameters of the assay, including Cas12a, crRNA, and the F-Q reporter, were systematically evaluated. The concentrations of Cas12a and crRNA were adjusted to achieve efficient enzyme-substrate complex formation, while the amount of the F-Q reporter was optimized to maximize signal intensity and minimize background fluorescence.

### 2.6 CRISPR/Cas12a and qPCR analysis of clinical samples

Cell-free DNA and RNA were isolated from serum samples using a serum/plasma cfDNA extraction kit and the miRcute serum/plasma miRNA isolation kit (Tiangen Biotech, China), respectively. For methodological validation, serum samples from 10 clinically confirmed TB patients were included as positive specimens, and 10 no-template controls (nuclease-free water, NTC) were used as negative controls to assess the ability of the assay to discriminate positive from negative samples. To evaluate the performance of the CRISPR/Cas12a platform in a clinical setting, all serum samples were first analyzed by the CRISPR/Cas12a fluorescence assay and then subjected to absolute quantification by qPCR as a reference method, allowing direct comparison of the analytical responses obtained by the two approaches.

Under the final reaction conditions established through the preceding sensitivity optimization experiments, four *tuberculosis*-associated targets, IS6110, gyrB, esxB, and ASdes, were analyzed in parallel. The clinical specimens used for CRISPR/Cas12a detection were identical to those subsequently subjected to qPCR analysis, ensuring direct comparability between the two methods. All targets were assayed within a unified core reaction framework. The CRISPR/Cas12a reaction mixture comprised LbCas12a protein, target-specific crRNA, 10 × CutSmart Buffer, an F-Q fluorescent reporter probe, and nucleic acid templates extracted from clinical samples. The amounts of Cas12a and the F-Q reporter probe were adjusted according to the target-specific optimization results, whereas all other components were kept constant. After assembly, 25 μL of each reaction mixture was transferred to a black 384-well plate and subjected to real-time fluorescence monitoring on a SpectraMax i3x microplate reader for 20 min under the optimized conditions. Fluorescence excitation and emission were set at 485 nm and 520 nm, respectively, with data collected at 1.5-min intervals. No-template controls were included in all assays to assess background signals. Endpoint fluorescence intensity at 20 min was used to classify samples as positive or negative and to compare the relative abundance of target nucleic acids among different clinical specimens.

To provide quantitative benchmarking and independent validation of the CRISPR/Cas12a results, qPCR was performed on the same clinical samples to detect and absolutely quantify *tuberculosis*-associated targets. qPCR assays were carried out using the HiScript II One Step qRT-PCR SYBR Green Kit. DNA targets (IS6110, esxB, and gyrB) were directly amplified in a 20 μL reaction volume, whereas the RNA target ASdes was analyzed using the same reaction system following a one-step reverse transcription-qPCR workflow. Each 20 μL reaction contained 1 μL of extracted DNA or RNA template, 10 μL of 2 × One Step SYBR Green Mix, 1 μL of One Step SYBR Green Enzyme Mix, 0.4 μL of 50 × ROX Reference Dye I, 0.4 μL of forward primer, and 0.4 μL of reverse primer, with nuclease-free water added to the final volume. Thermal cycling conditions were as follows: reverse transcription at 50 °C for 3 min, initial denaturation at 95 °C for 3 s, followed by 40 cycles of 95 °C for 10 s and 60 °C for 30 s. A melting curve analysis was performed after amplification to confirm product specificity. All reactions, including no-template controls, were run in triplicate, and Ct values were calculated as the mean of the three technical replicates. For absolute quantification, serial dilutions of four synthetic MTB oligonucleotide standards with known concentrations were used to generate standard curves for IS6110, esxB, gyrB, and ASdes. Target nucleic acid levels in clinical samples were calculated based on the corresponding standard curves. Primer sequences for each target are listed in Table 1.

### 2.7 Construction of an integrated FTA card-based ERA/RT-ERA–CRISPR/Cas12a workflow for lateral flow detection

Fifteen microliters of clinical serum or control samples were applied onto a 2 × 4 mm area of a FTA card (Cytiva, formerly GE Healthcare, USA) and allowed to dry at room temperature in the dark. The selected 2 × 4 mm sampling area was determined based on the absorption capacity of the FTA matrix and the requirement for sufficient nucleic acid recovery while minimizing reagent consumption. This defined area was sufficient to accommodate the applied sample volume (15 μL) and provided consistent nucleic acid release for downstream ERA/RT-ERA amplification. After drying, the FTA card was transferred to a 1.5 mL RNase-free microcentrifuge tube, followed by the addition of 200 μL nuclease-free water. The tube was incubated at room temperature for 5 min to obtain the FTA card eluate. Although this elution procedure introduces an approximately 100-fold dilution relative to the original eluate volume (200 μL elution volume, with 2 μL eluate used as amplification template), this potential loss of target concentration was compensated by the high amplification efficiency of ERA/RT-ERA, which enables robust enrichment of low-abundance DNA and RNA targets under optimized isothermal conditions. It should be noted that the FTA card elution procedure introduces an approximately 100-fold dilution relative to the original sample volume (200 μL elution volume, with 2 μL eluate used as the amplification template), which may reduce the effective concentration of target nucleic acids. This potential reduction in target concentration was addressed by the high amplification efficiency of ERA/RT-ERA, which enables sensitive amplification of low-copy targets under optimized isothermal conditions. Following FTA card-mediated nucleic acid release, the eluate was directly used as the template for subsequent ERA (DNA targets) or RT-ERA (RNA targets) amplification without additional column-based or magnetic bead-based purification. ERA or RT-ERA reaction was carried out in a total volume of 48 μL, consisting of 20 μL ERA solvent, 0.5 μL forward primer, 2.5 μL reverse primer, 21 μL ddH_2_O, and 2 μL FTA card eluate. The reaction mixture was then transferred into a reaction tube containing ERA lyophilized reagents and mixed thoroughly. Subsequently, 2 μL ERA activator was added to the inner side of the tube cap, followed by rapid sealing and brief centrifugation. The reaction was incubated at 42 °C for 20 min to complete the ERA or RT-ERA isothermal amplification.

Upon completion of ERA or RT-ERA amplification, 1 μL of the amplicon was directly subjected to CRISPR/Cas12a-based lateral flow detection. The CRISPR/Cas12a reaction was performed under previously optimized conditions in a total volume of 100 μL, comprising Cas12a protein, a target-specific crRNA, an F-B-labeled reporter probe, and CutSmart Buffer at a final concentration of 1 × . The reaction mixture was incubated at 37 °C for 15 min to activate the trans-cleavage activity of Cas12a. Following incubation, 50 μL of the reaction mixture was applied to the sample pad of a lateral flow strip (TS104; Suzhou Xinda Gene Technology Co., Ltd., Suzhou, China). Results were visually interpreted within 10 min: the simultaneous appearance of both the test line (T) and control line (C) was considered positive, whereas the presence of only the control line indicated a negative result.

### 2.8 Data analysis

All experiments and assays were performed in at least three independent replicates. Data are presented as mean ± standard deviation (SD). Statistical analysis and data visualization were carried out using GraphPad Prism 8.0.

### 2.9 Statistical analysis

The limit of detection (LOD) for each target was calculated using the 3σ method, defined as the target concentration at which the mean signal exceeded the mean signal of the no-template control (NTC) plus three times the standard deviation of the NTC. For each target, LOD determination was based on at least three independent replicates (*n* ≥ 3); the mean and standard deviation used for the 3σ threshold were likewise derived from these replicate measurements of the NTC. qPCR standard curves were constructed by plotting Ct values against the logarithm of template copy number for each target; the linear range, slope, and coefficient of determination (*R*^2^) were reported for each standard curve. A sample was classified as positive in the CRISPR/Cas12a fluorescence assay when its endpoint fluorescence signal exceeded the mean NTC signal plus 3σ. For lateral flow strips, a sample was classified as positive when a visible test line was observed alongside the control line. Concordance between CRISPR/Cas12a results (fluorescence and lateral flow) and qPCR was assessed by comparing qualitative calls (positive/negative) and the relative abundance trends across samples.

## 3 Results

### 3.1 Establishment, optimization, and clinical validation of a CRISPR/Cas12a fluorescence detection platform targeting IS6110

To assess the performance of the CRISPR/Cas12a detection module within a conventional molecular diagnostic workflow, serum samples from patients with tuberculosis were collected and subjected to either DNA or RNA extraction. The recovered nucleic acids were used as templates for PCR or RT-PCR amplification to generate double-stranded DNA corresponding to the target sequences, and the resulting amplicons were then directly analyzed using the CRISPR/Cas12a fluorescence assay. In the detection reaction, crRNA-directed Cas12a specifically recognizes the target dsDNA, which activates its trans-cleavage function and leads to non-specific cleavage of a fluorescently labeled single-stranded reporter, thereby producing a measurable fluorescence signal. Fluorescence output was recorded in real time using a microplate reader to characterize the assay response to target nucleic acids (Figure 1A). To ensure consistency and quality of the core enzymatic component, recombinant Cas12a protein was expressed, purified, and its purity verified by SDS-PAGE (Supplementary Figure S1).

**Figure 1:**
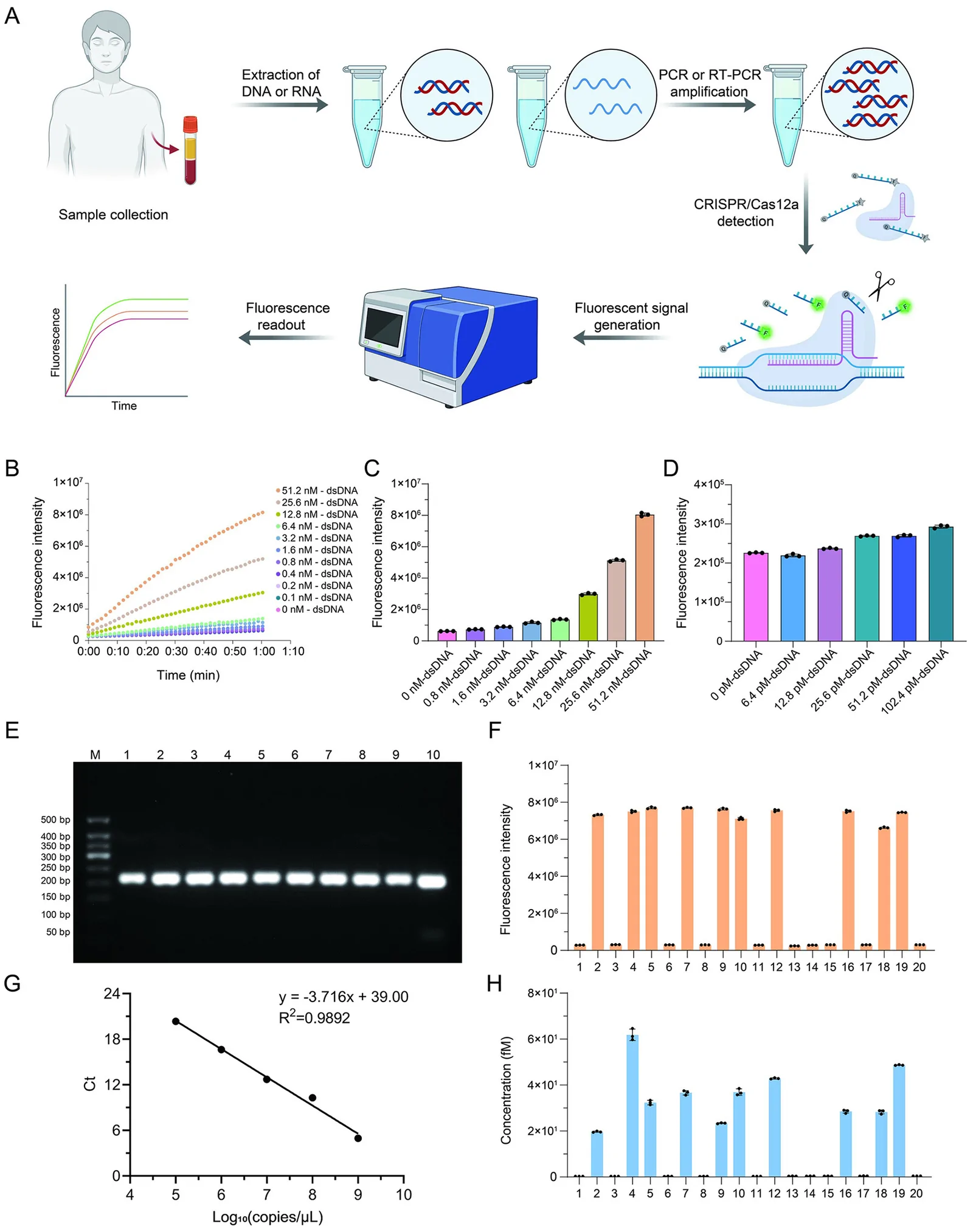
Construction, optimization, and clinical validation of the IS6110-based CRISPR/Cas12a fluorescence detection system. **(A)** Schematic representation of the CRISPR/Cas12a fluorescence detection module within a conventional molecular diagnostic workflow. Serum samples were subjected to DNA or RNA extraction, followed by PCR or RT-PCR amplification. The resulting double-stranded DNA products served as inputs to trigger Cas12a trans-cleavage activity, generating a fluorescence signal. **(B)** Real-time fluorescence kinetics of the IS6110 CRISPR/Cas12a assay at varying dsDNA template concentrations, including a no-template control (NTC). **(C)** Initial analytical sensitivity of the IS6110 CRISPR/Cas12a assay determined based on endpoint fluorescence signals. **(D)** Optimized IS6110 CRISPR/Cas12a fluorescence detection after increasing Cas12a protein concentration, showing improved sensitivity. **(E)** Agarose gel electrophoresis of PCR-amplified IS6110 from DNA extracted from clinical serum samples (amplicon size: 208 bp). **(F)** Endpoint fluorescence signals of the IS6110 target in clinical serum samples measured by CRISPR/Cas12a. **(G)** qPCR standard curve generated using serial dilutions of a synthetic IS6110 template. **(H)** Quantification of IS6110 copy numbers in clinical serum samples based on the qPCR standard curve.

To evaluate the feasibility and analytical performance of the CRISPR/Cas12a system for detecting Mtb nucleic acids, the multicopy repetitive element IS6110 was selected as the initial target. A Cas12a-based fluorescence assay was successfully established, and its reaction characteristics were examined by real-time fluorescence monitoring. Upon addition of IS6110 dsDNA at different concentrations, a rapid and time-dependent increase in fluorescence was observed at the early stage of the reaction. In contrast, the NTC remained at baseline throughout the assay (Figure 1B), demonstrating that the assay could effectively distinguish target nucleic acids from the negative control and generate significantly different fluorescence signals across varying template concentrations. Based on the lowest template concentration that could be reliably distinguished from the NTC at the endpoint, the initial assay exhibited a limit of detection (LOD) of 0.78 nM (Figure 1C). Here, the analytical sensitivity evaluation was performed using PCR-generated double-stranded IS6110 amplicons as target substrates and was intended to characterize the detection capability of the CRISPR/Cas12a fluorescence module rather than the final sensitivity of the integrated ERA amplification workflow. Although the preliminary system already demonstrated reliable detectability, there remained room for improving sensitivity. Therefore, the Cas12a concentration was further optimized as an important reaction parameter by increasing its final concentration from 250 nM to 300 nM. Under the optimized conditions, the assay generated an enhanced fluorescence response within approximately 20 min at 37 °C (Figure 1D). Notably, the apparent detection limit of the CRISPR/Cas12a fluorescence module was reduced from 0.78 nM to 12.5 pM, representing an approximately 62-fold improvement in sensitivity relative to the initial configuration, together with a marked reduction in assay time. Collectively, these results demonstrate the successful establishment and optimization of an IS6110-based CRISPR/Cas12a fluorescence detection platform with high feasibility and sensitivity.

To evaluate the applicability and stability of the established CRISPR/Cas12a fluorescence detection platform in clinical settings, we first performed CRISPR/Cas12a-based detection of the IS6110 target in serum samples. In parallel, synthetic nucleic acids corresponding to each target were serially diluted to generate qPCR standard curves, which were then used to quantify the same set of clinical samples by real-time qPCR. The quantitative outputs obtained by the two methods were subsequently compared to evaluate the consistency of their response patterns in clinical specimens.

For the DNA target IS6110, genomic DNA extracted from serum yielded clear and specific amplicons following PCR amplification (Figure 1E). In the IS6110-CRISPR/Cas12a fluorescence assay, all positive samples produced fluorescence signals markedly higher than those of the negative controls, with substantial signal heterogeneity observed across individual samples (Figure 1F). For quantitative reference, a 10-fold serial dilution of a synthetic IS6110 template was used to construct a qPCR standard curve, which showed good linearity (*R*^2^ = 0.9892) (Figure 1G). qPCR analysis of the same clinical serum samples revealed pronounced differences in IS6110 copy numbers (Figure 1H), and these variations closely mirrored the trends observed in the CRISPR/Cas12a fluorescence readouts, indicating strong concordance between the two detection modalities.

### 3.2 Construction and validation of an integrated FTA-ERA/RT-ERA-CRISPR/Cas12a workflow for FTA card-based detection of blood nucleic acids

To enable rapid and straightforward detection of MTB nucleic acids in blood, we established an integrated workflow combining FTA cards, ERA/RT-ERA isothermal amplification, and CRISPR/Cas12a lateral flow readout, bypassing conventional nucleic acid extraction. The overall strategy is illustrated in Figure 2A. By directly applying finger-prick blood or clinical serum samples onto an FTA card, nucleic acids can be rapidly captured and stably preserved without complex pretreatment. Following a brief wash and incubation with water, the released nucleic acids can serve directly as templates for downstream amplification, eliminating the need for specialized equipment or labor-intensive extraction procedures. As shown in Figure 2B, the FTA card remained structurally intact after sample application and air-drying, with the sample adsorption area clearly defined, making it suitable for subsequent elution. To assess whether nucleic acids processed via FTA retained integrity and amplifiability, the eluates were used as templates for PCR amplification of the MTB-specific target IS6110, followed by agarose gel electrophoresis. Specific amplification bands of the expected size were observed in the corresponding lanes (Figure 2C), indicating that FTA card processing did not noticeably compromise nucleic acid integrity and that the eluates were suitable for direct use in molecular amplification reactions.

**Figure 2:**
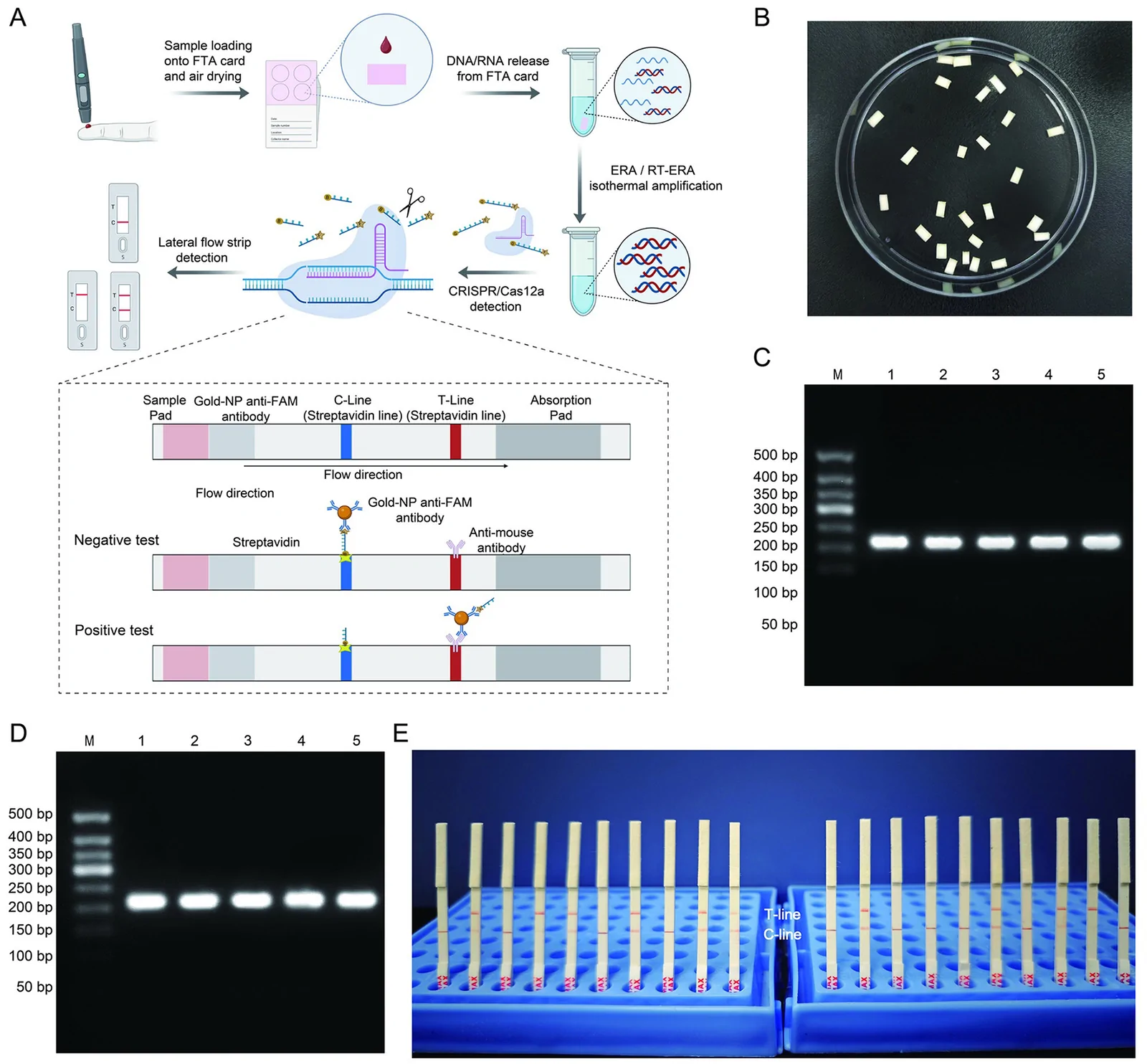
Construction and validation of FTA-ERA/RT-ERA-CRISPR/Cas12a workflow for blood nucleic acids based on the IS6110 target. **(A)** Schematic of the integrated FTA-ERA/RT-ERA-CRISPR/Cas12a workflow for FTA card-based detection of IS6110. **(B)** Appearance of an FTA card after application and drying of clinical serum samples, showing the area for adsorption and subsequent elution of IS6110 target nucleic acids. **(C)** Agarose gel electrophoresis of PCR-amplified IS6110 using FTA card eluates as templates. The expected amplicon size is 208 bp. **(D)** Gel electrophoresis analysis of ERA-amplified IS6110 products using FTA card eluates as templates. The amplicon size is 208 bp. **(E)** Lateral flow readout of the CRISPR/Cas12a assay using ERA-amplified IS6110 products. Positive and negative calls for samples 1-20 were consistent with fluorescence and qPCR results.

Building on this approach, we further evaluated the feasibility of using FTA card eluates as templates for ERA isothermal amplification. Eluates from the same series of clinical samples were used as input, and the resulting ERA products were analyzed by gel electrophoresis. As shown in Figure 2D, all samples produced clear and specific amplification signals without detectable non-specific bands, indicating that the FTA-ERA system can efficiently amplify the IS6110 target under isothermal conditions, providing sufficient substrate for subsequent CRISPR/Cas12a detection. The ERA products were then directly applied to the CRISPR/Cas12a lateral flow detection module for analysis of the same clinical samples. As illustrated in Figure 2E, positive samples displayed distinct bands at both the test (T) and control (C) lines, whereas negative samples showed only the C line, allowing straightforward visual interpretation. Notably, the lateral flow results were consistent with those obtained from the fluorescence detection system (**Figures 1F, H**), further confirming the reliability and reproducibility of the FTA-ERA/RT-ERA-CRISPR/Cas12a workflow across different signal output modes. In summary, we established a nucleic acid detection workflow suitable for blood samples. This integrated approach combines sample processing, isothermal amplification, and CRISPR/Cas12a lateral flow detection, maintaining high specificity and sensitivity while significantly simplifying the procedure. It provides a practical solution for rapid on-site screening of tuberculosis and application in resource-limited settings.

### 3.3 Performance evaluation of the single-copy target esxB in the CRISPR/Cas12a assay and validation of the integrated workflow

Following the establishment of the platform for the multi-copy sequence IS6110, we further extended the CRISPR/Cas12a detection system to the single-copy MTB DNA target esxB to evaluate its performance under low-copy-number conditions. Real-time fluorescence kinetics demonstrated that under 37 °C, double-stranded esxB DNA effectively activated Cas12a trans-cleavage activity, producing fluorescence signals that accumulated over time. The signal growth rate correlated positively with template concentration (Figure 3A), indicating a clear concentration-dependent response even for this single-copy target. Using PCR-generated esxB dsDNA amplicons as substrates for Cas12a detection, the analytical sensitivity of the fluorescence module was initially evaluated, with a detection limit of 1.56 nM (Figure 3B). Subsequently, key reaction parameters were optimized. The concentration of the fluorescent reporter probe was found to significantly influence background signal. Reducing the F-Q reporter concentration likely decreased the background arising from non-specific probe degradation or direct Cas12a-substrate interaction, thereby improving the signal-to-noise ratio without compromising the specific signal amplitude. Under these optimized conditions, reducing the final concentration of the F–Q probe from 1 μM to 833 nM substantially decreased non-specific background fluorescence while maintaining the amplitude of the specific signal, lowering the detection limit of the CRISPR/Cas12a fluorescence module to 6.25 pM (Figure 3C), representing an approximately 250-fold improvement in sensitivity compared to the initial setup.

**Figure 3:**
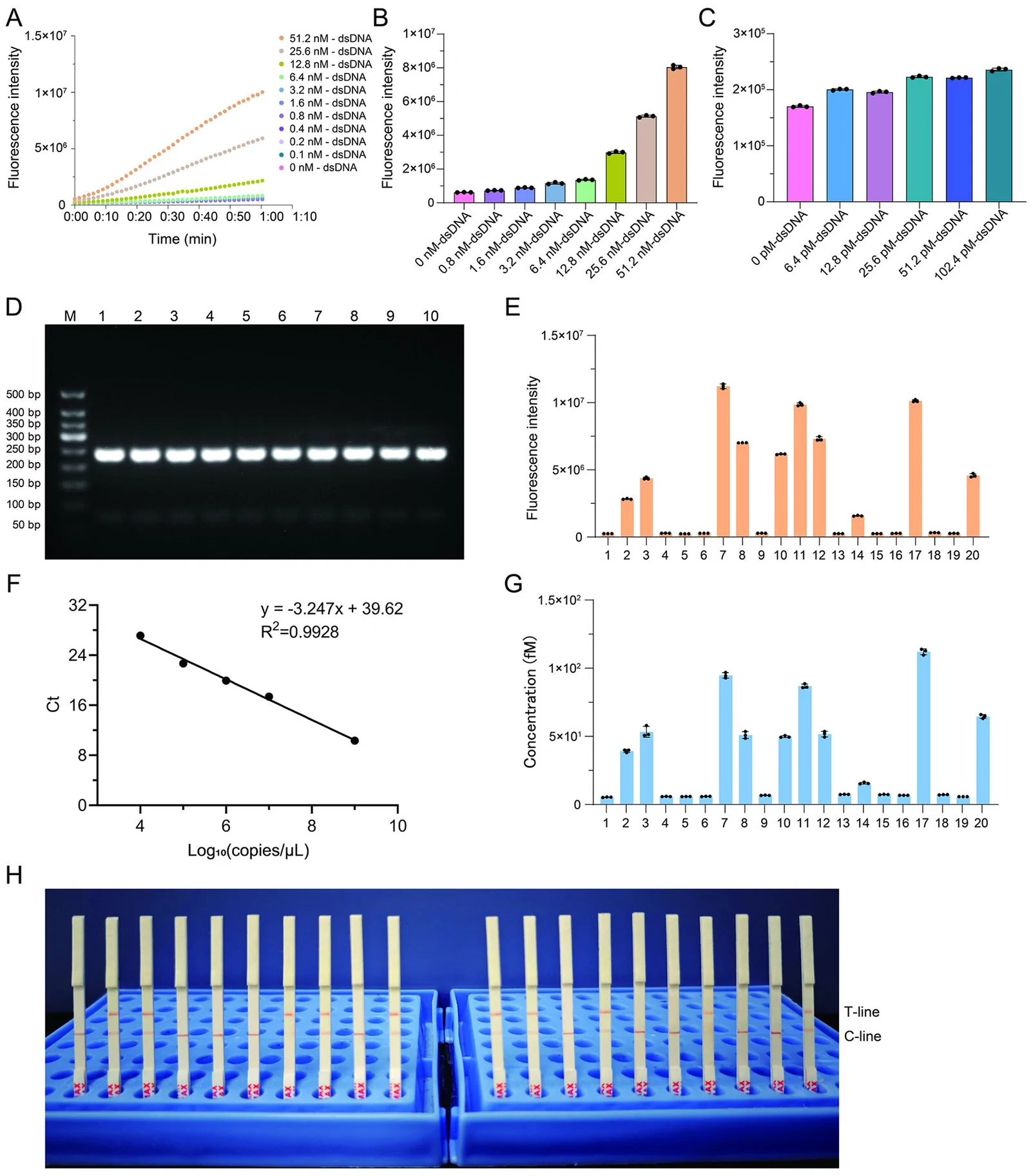
Performance of CRISPR/Cas12a detection for the single-copy *M. tuberculosis* target esxB. **(A)** Real-time fluorescence kinetics of CRISPR/Cas12a with varying concentrations of double-stranded esxB DNA at 37 °C. **(B)** Analytical sensitivity of the initial esxB CRISPR/Cas12a detection system. **(C)** Improved sensitivity of the esxB CRISPR/Cas12a assay after adjustment of the F-Q reporter probe concentration. **(D)** Agarose gel electrophoresis of PCR-amplified esxB from serum-derived cfDNA (amplicon size: 236 bp). **(E)** Endpoint fluorescence signals of esxB amplification products from clinical samples measured by CRISPR/Cas12a. **(F)** qPCR standard curve constructed using serial dilutions of a synthetic esxB DNA template. **(G)** Absolute quantification of esxB copy numbers in clinical samples based on the qPCR standard curve. **(H)** Lateral flow readout of esxB detection using the integrated FTA-ERA/RT-ERA-CRISPR/Cas12a FTA card-based workflow. Positive and negative calls for samples 1–20 were consistent with results shown in panels E and G.

Clinical validation of the esxB target was then performed using serum-derived cfDNA. Following PCR amplification, agarose gel electrophoresis revealed a single band of the expected size for all positive samples (Figure 3D). The amplified products were subsequently subjected to CRISPR/Cas12a fluorescence detection, in which the endpoint fluorescence signals of all positive samples were markedly higher than those of the negative controls. Notably, signal intensities varied substantially between individual patient samples (Figure 3E), indicating significant inter-individual differences in circulating MTB DNA abundance. To provide quantitative reference, a qPCR standard curve was constructed using a synthetic esxB template, showing excellent linearity (*R*^2^ = 0.9928; Figure 3F). Absolute quantification of the same clinical samples (samples 1–10, identical to those analyzed in section 3.1) based on this standard curve revealed pronounced differences in esxB copy numbers across individuals (Figure 3G). Importantly, the relative abundance trends observed among samples were highly consistent with the CRISPR/Cas12a fluorescence readouts, demonstrating that this detection system not only enables reliable positive identification but also reflects relative variations in target nucleic acid abundance within a certain range. The quantitative concordance between CRISPR/Cas12a fluorescence and qPCR was confirmed across all analyzed samples.

Building on these results, we further evaluated the suitability of the esxB target within the FTA-ERA/RT-ERA-CRISPR/Cas12a workflow. Clinical serum samples (samples 1–20, comprising the same set used for integrated workflow validation) were applied to FTA cards and eluted, and the resulting eluates were directly used as templates for ERA amplification. The amplified products were then introduced into the Cas12a lateral flow detection module. As shown in Figure 3H, positive samples produced clear bands at both the test (T) and control (C) lines, whereas negative controls exhibited only the C line. The readout was consistent with the fluorescence detection mode and qPCR-based quantification. These results indicate that even for a single-copy DNA target, the coupled FTA-ERA/RT-ERA and CRISPR/Cas12a system can provide stable and reliable visual output.

### 3.4 Performance evaluation of the single-copy target gyrB in the CRISPR/Cas12a assay and validation of the integrated workflow

Following validation of the multi-copy target IS6110 and the single-copy target esxB, we further assessed the generalizability of the detection strategy across targets with different gene copy numbers by extending the workflow to the single-copy conserved gene gyrB. Under 37 °C reaction conditions, double-stranded gyrB DNA effectively activated Cas12a trans-cleavage activity, producing fluorescence signals that accumulated over time. Clear differences in signal intensity were observed among varying template concentrations (Figure 4A), indicating a robust concentration-dependent response for this target as well. Under the initial reaction conditions, the analytical sensitivity of the CRISPR/Cas12a fluorescence module using PCR-generated gyrB dsDNA amplicons was initially evaluated, resulting in an LOD of 0.1 nM (Figure 4B). Optimization of the reaction system was subsequently performed by adjusting the Cas12a protein concentration. Increasing the final Cas12a concentration from 250 nM to 300 nM improved the reaction kinetics and enhanced sensitivity, lowering the LOD to 25 pM (Figure 4C). These findings are consistent with the optimization trends observed for the previous targets, confirming that the effective concentration of Cas12a influences trans-cleavage efficiency and overall assay sensitivity under the tested experimental conditions.

**Figure 4:**
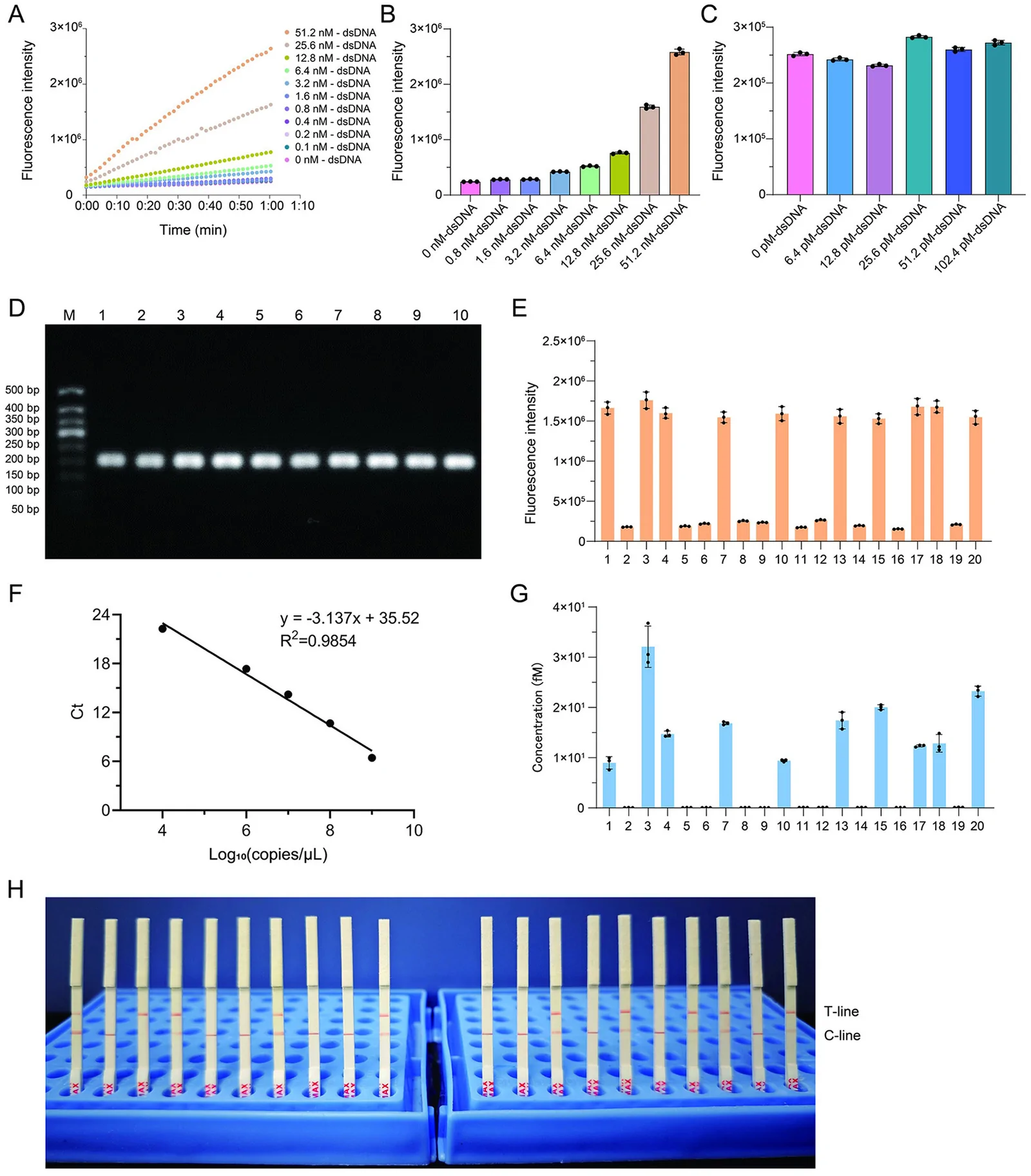
Fluorescence performance of CRISPR/Cas12a for the gyrB target and its application in the integrated FTA-ERA/RT-ERA-CRISPR/Cas12a workflow. **(A)** Real-time fluorescence kinetics of CRISPR/Cas12a triggered by double-stranded gyrB DNA at varying concentrations under 37 °C. **(B)** Analytical sensitivity assessment and LOD determination of the initial gyrB CRISPR/Cas12a detection system. **(C)** Improved detection performance and sensitivity of the optimized system following an increase in the final Cas12a concentration. **(D)** Agarose gel electrophoresis of PCR-amplified gyrB from serum-derived cfDNA (amplicon size: 198 bp). **(E)** Endpoint fluorescence signals of gyrB in clinical samples measured by CRISPR/Cas12a. **(F)** qPCR standard curve generated using serial dilutions of a synthetic gyrB template. **(G)** Absolute quantification of gyrB in the same batch of clinical serum samples based on the qPCR standard curve. **(H)** Lateral flow readout of gyrB detection in clinical samples using the integrated FTA-ERA/RT-ERA-CRISPR/Cas12a FTA card-based workflow. Positive and negative calls for samples 1–20 were consistent with results shown in panels E and G.

Clinical validation of the gyrB target was subsequently performed. Serum-derived cfDNA from the same clinical sample set (samples 1–10) was amplified by PCR, yielding a single, specific band of the expected size on agarose gel electrophoresis (Figure 4D). The PCR products were then subjected to the gyrB CRISPR/Cas12a fluorescence assay, where endpoint fluorescence signals of positive samples were markedly higher than those of negative controls (Figure 4E). For quantitative reference, a qPCR standard curve was generated using a synthetic gyrB template, exhibiting strong linearity (*R*^2^ = 0.9854; Figure 4F). Absolute quantification of the same clinical samples based on this curve revealed trends consistent with the CRISPR/Cas12a fluorescence readouts (Figure 4G), confirming strong quantitative concordance between the two methods.

Building on this, eluates from FTA card-treated serum samples (samples 1–20) were directly used as templates for ERA amplification, and the amplified products were introduced into the CRISPR/Cas12a lateral flow module for visual detection to assess the suitability of the gyrB target in the FTA-ERA/RT-ERA-CRISPR/Cas12a workflow. As shown in Figure 4H, positive samples produced clear bands at both the test (T) and control (C) lines, whereas negative controls exhibited only the C line. The lateral flow results were consistent with both the fluorescence readouts (Figure 4E) and qPCR quantification (Figure 4G), demonstrating that the integrated FTA card-based workflow is capable of stable detection of the single-copy gyrB target and maintains reliable performance across different signal output modes.

### 3.5 Construction of the CRISPR/Cas12a assay for the rna target asdes and validation in the integrated workflow

After establishing detection systems for multiple DNA targets, we extended the strategy to the RNA target ASdes to evaluate the platform's applicability across different nucleic acid types. Under 37 °C reaction conditions, the Cas12a system activated by double-stranded ASdes DNA generated fluorescence signals that accumulated over time, showing a stable concentration-dependent response across different template concentrations (Figure 5A). Using PCR-generated ASdes double-stranded DNA products as substrates, the CRISPR/Cas12a fluorescence module showed an initial analytical detection limit of 0.1 nM.(Figure 5B). Increasing the final Cas12a concentration from 250 nM to 300 nM significantly enhanced the trans-cleavage reaction rate, improving system sensitivity and lowering the LOD to 12.5 pM (Figure 5C). This optimization trend was consistent with the observations for DNA targets, indicating that the effective Cas12a concentration is a critical determinant of both reaction kinetics and assay sensitivity. Clinical validation was then performed using serum-derived ASdes RNA from the same clinical sample set (samples 1–10). Following cfRNA extraction and RT-PCR amplification, agarose gel electrophoresis revealed a single, clear, specific band (Figure 5D), confirming the high specificity of the amplification system. The amplified products were subsequently tested in the ASdes CRISPR/Cas12a fluorescence assay, in which endpoint signals of positive samples were markedly higher than the no-template controls, allowing reliable discrimination between positive and negative samples (Figure 5E).

**Figure 5:**
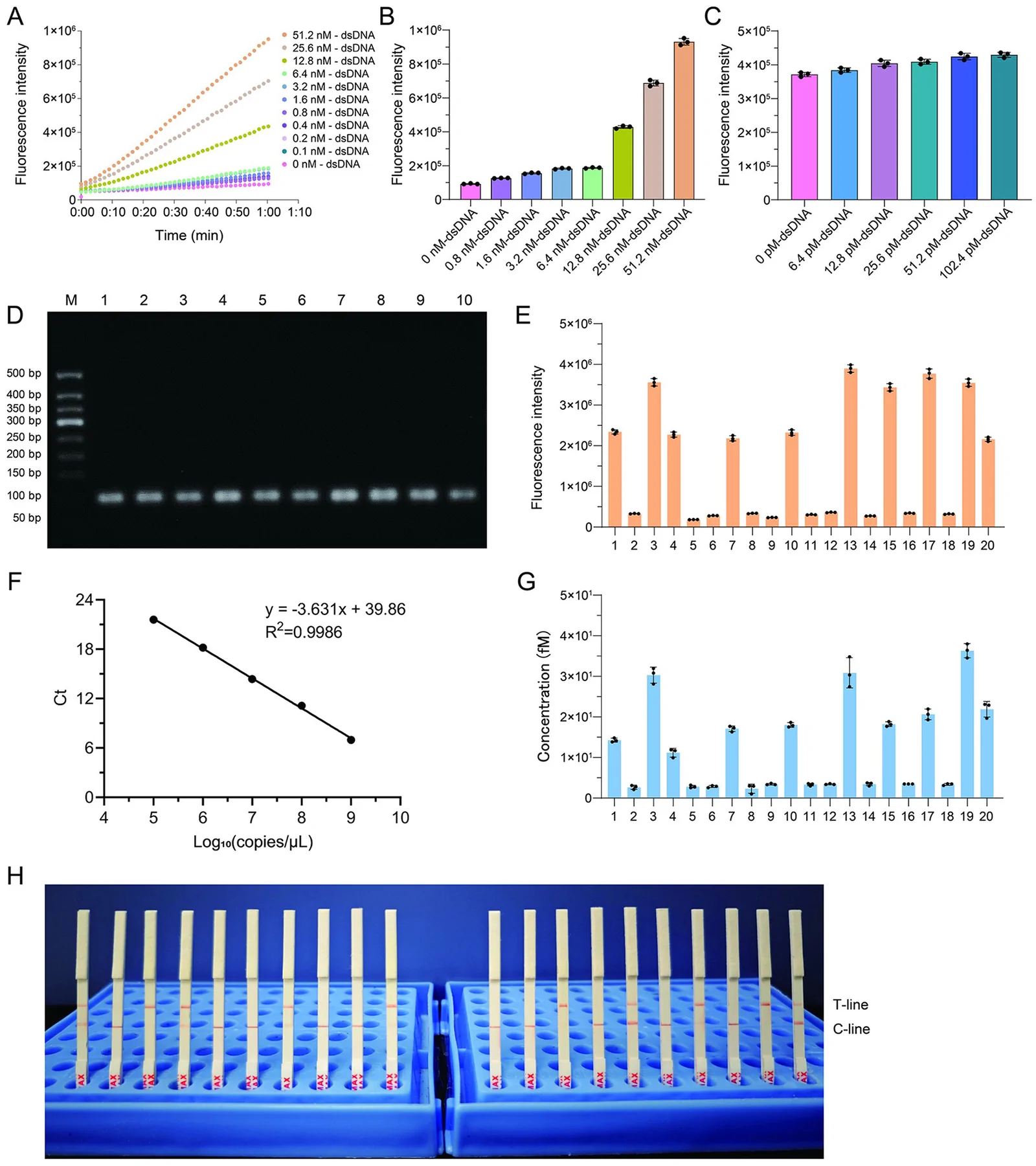
Construction of the CRISPR/Cas12a detection system for the RNA target ASdes and validation of the integrated FTA card-based workflow. **(A)** Real-time fluorescence kinetics of CRISPR/Cas12a activated by ASdes amplification products at different concentrations under 37 °C. **(B)** Analytical sensitivity assessment and determination of the detection limit (LOD) for the initial ASdes-CRISPR/Cas12a assay. **(C)** Enhanced sensitivity of the assay after increasing the final Cas12a concentration to 300 nM. **(D)** Agarose gel electrophoresis of RT-PCR products from clinical serum-derived cfRNA, showing a specific ASdes amplification band. **(E)** Endpoint fluorescence detection of the ASdes target in clinical samples. **(F)** Real-time qPCR standard curve constructed using a synthetic ASdes template. **(G)** Absolute quantification of ASdes RNA in the same batch of clinical serum samples based on the qPCR standard curve. **(H)** Lateral flow readout of the integrated FTA-RT-ERA-CRISPR/Cas12a FTA card-based workflow. Samples identified as positive by fluorescence showed clear bands at both the test **(T)** and control **(C)** lines, whereas negative controls displayed only the C line. Results were consistent with fluorescence detection and qPCR quantification.

To provide quantitative reference, a qPCR standard curve was generated using a synthetic ASdes template, exhibiting excellent linearity (*R*^2^ = 0.9986; Figure 5F). Absolute quantification of the same clinical samples based on this curve revealed notable differences in ASdes copy numbers across individuals, with trends closely matching the CRISPR/Cas12a fluorescence readouts (Figure 5G), further validating the system's accuracy for RNA quantification and confirming strong quantitative concordance between methods.

Building on these results, we integrated the detection workflow into an FTA-RT-ERA-CRISPR/Cas12a platform to evaluate the detectability of ASdes RNA under simplified sample-processing conditions. Clinical serum samples (samples 1–20) were applied to FTA cards, followed by lysis and elution. The eluates were directly used as RT-ERA templates, and the resulting amplification products were subsequently analyzed in the Cas12a lateral flow module for visual readout. All samples that tested positive by fluorescence displayed clear bands at both the test (T) and control (C) lines, whereas negative controls showed only the C line (Figure 5H). The lateral flow results were fully consistent with fluorescence intensity (Figure 5E) and qPCR-based quantification (Figure 5G), demonstrating that the integrated FTA card-based workflow provides reliable readout consistency for RNA targets.

To facilitate comparison with previously reported CRISPR-based diagnostic platforms, the molar concentrations of the analytical detection limits were converted into absolute copy numbers according to the molecular weight of the corresponding double-stranded DNA amplicons (660 g/mol per base pair). The calculated detection limits were approximately 193 copies/μL for IS6110 (59 bp), 97 copies/μL for esxB (59 bp), 387 copies/μL for gyrB (59 bp), and 116 copies/μL for ASdes (98 bp). These values represent the analytical sensitivity of the CRISPR/Cas12a fluorescence detection module using amplified DNA substrates. Taken together with the results obtained for IS6110, esxB, and gyrB, these findings demonstrate that the CRISPR/Cas12a detection framework can accommodate multiple Mtb-associated nucleic acid targets with different genomic characteristics and nucleic acid types. However, these targets were evaluated individually in the present study, and their combined diagnostic performance requires further validation using larger clinical cohorts and parallel multiple distinct targets testing. Both fluorescence readouts and lateral flow visual outputs closely reflect the trends observed by qPCR, confirming that this system can reliably capture target abundance differences in complex clinical serum matrices and provides a solid technical foundation for developing multiple distinct targets molecular diagnostics suitable for point-of-care applications.

## 4 Discussion

Rapid, sensitive, and field-deployable molecular detection of Mtb remains a formidable challenge, particularly in resource-limited settings (Chang et al., 2022; Huang et al., 2025). To address this need, we developed an integrated platform that combines FTA card-based simplified sample processing, ERA/RT-ERA isothermal amplification, and CRISPR/Cas12a signal detection with dual-mode readout. The FTA card approach releases and stabilizes nucleic acids at room temperature, avoiding conventional column- or magnetic bead-based extraction and thereby reducing dependence on cold-chain logistics, specialized equipment, and trained personnel. Coupled with isothermal amplification, which proceeds at a constant temperature without thermal cycling, and with Cas12a-mediated signal generation, the workflow enables both quantitative fluorescence analysis and instrument-free visual interpretation via lateral flow strips. Across the four targets tested, the fluorescence and lateral flow readouts showed trends consistent with qPCR, supporting the analytical feasibility of the integrated platform.

An important feature of this study is a multiple distinct targets design that spans genomic and transcription-associated signals, providing complementary information beyond single-target CRISPR/Cas12a assays. The targets were selected to balance three characteristics, genomic copy number, sequence conservation, and transcriptional activity, given that circulating Mtb-derived cfDNA/cfRNA in blood is present at extremely low abundance and is heavily interfered with by host nucleic acid background. IS6110, a multicopy insertion element (typically 6–25 copies per genome), consistently yielded the highest analytical sensitivity among the four targets, as expected from the greater number of available molecular targets (Adugna et al., 2026; van Dongen and Segerink, 2025). However, reliance on IS6110 alone carries a risk of false-negative results, because certain strains, particularly those circulating in parts of Southeast Asia, harbor zero or very few copies. Two single-copy chromosomal targets, gyrB and esxB, were therefore included as conserved markers that provide redundancy independent of IS6110 copy-number variation; their distinct sensitivity profiles after optimization suggest that some genomic loci are more amenable to ERA amplification or crRNA-directed Cas12a recognition, an observation meriting further study. The RNA target ASdes adds a transcription-associated dimension: Mtb-derived small RNAs, including ASdes, have been detected in plasma from patients with active tuberculosis (Han et al., 2021; Bagi et al., 2025; Du et al., 2025), and may reflect actively replicating bacteria rather than merely genomic presence. However, whether circulating ASdes levels accurately reflect bacterial burden, disease progression, or treatment response remains to be established. It should be noted that the four targets were evaluated independently rather than measured simultaneously within the same specimen; therefore, target concordance rates within individual patients, and any predefined diagnostic interpretation rule, could not be determined. Future studies with parallel detection of all targets in the same specimens will be required to define optimal target combinations and clinically applicable interpretation algorithms (Hui et al., 2025).

A systematic comparison with recently reported CRISPR/Cas12a-based TB detection platforms (Supplementary Table S2) highlights the features of the present system. Gan et al. (2024) developed an ERA-CRISPR/Cas12a platform targeting IS6110 alone, but lacked the multiple distinct targets breadth and RNA coverage of our platform (Gan et al., 2024). Jia et al. (2023) reported a sensitive CRISPR/Cas12a-based TB assay, yet still required conventional nucleic acid extraction and relied exclusively on fluorescence readout (Jia et al., 2023). Bell et al. (2025) described a streamlined CRISPR-based test for direct sputum detection without extraction, published in Science Advances, but this platform was designed for a single target without multiple distinct targets redundancy or dual-mode readout (Bell et al., 2025). In comparison, the present platform offers several operational advantages: FTA card-based processing removes conventional extraction steps; the integrated workflow completes in approximately 45–60 min; and dual-mode readout supports both quantitative fluorescence and instrument-free lateral flow interpretation (Shang et al., 2024). A formal cost-effectiveness analysis was, however, not performed and remains to be evaluated with respect to reagent consumption, labor, and large-scale implementation scenarios (Chen et al., 2024).

Several limitations of this study should be acknowledged. First, the clinical cohort was small (31 samples) and consisted exclusively of tuberculosis-positive specimens (8 culture-positive and 23 molecular-testing-positive), with only a small number of commercially obtained normal serum samples used as negative matrix controls. Clinical negative samples, healthy individuals, smear-negative TB patients, or patients with other pulmonary diseases such as non-tuberculous mycobacterial infection, were not included. Consequently, this work is a preliminary feasibility assessment rather than a comprehensive diagnostic accuracy study, and clinical specificity, false-positive rates, and differential diagnostic performance could not be evaluated. Second, the reported limits of detection reflect the analytical sensitivity of the CRISPR/Cas12a fluorescence detection module using amplified substrates, rather than the final sensitivity of the complete FTA-ERA/RT-ERA-CRISPR/Cas12a workflow; the comparatively high Cas12a concentration (300 nM) used to improve sensitivity may also increase reagent consumption and background, and warrants further optimization of enzyme loading, reaction kinetics, and background suppression. Third, the FTA elution procedure introduces an approximately 100-fold dilution (200 μL elution with 2 μL used as template), which may reduce the availability of ultra-low-abundance circulating nucleic acids and increase false-negative risk; although ERA/RT-ERA amplification and Cas12a signal amplification provide multiple levels of enhancement, a direct FTA-treated vs. non-FTA-treated comparison was not performed, so the quantitative impact of this dilution on the limit of detection remains to be determined. Fourth, the analysis was based on serum rather than sputum, the primary specimen for routine TB diagnosis, and sputum's higher viscosity, complex microbial composition, and abundant host components may affect nucleic acid release, amplification, and background; adaptation to sputum will require further pretreatment optimization and matrix validation. Finally, analytical specificity against closely related mycobacteria (e.g., *M. bovis, M. avium, M. kansasii*) and common respiratory pathogens was not systematically evaluated, and a systematic comparison with mycobacterial culture was not performed (Jia et al., 2023; Rafał et al., 2025). Future studies incorporating larger, prospective cohorts with both TB-positive and TB-negative samples, standardized reference methods, cross-reactivity panels, and parallel detection of all targets will be necessary to establish the diagnostic accuracy and specificity of this platform (Xiao et al., 2023; Lin et al., 2025).

In summary, this study demonstrates the feasibility of a FTA-ERA/RT-ERA-CRISPR/Cas12a platform for detecting low-abundance Mtb DNA and RNA signals in serum without conventional nucleic acid extraction, balancing sensitivity, target diversity, and operational simplicity. As a preliminary feasibility assessment, it lays a foundation for future sputum-based testing, expanded clinical validation, and comprehensive specificity evaluation toward point-of-care TB diagnosis.

## Data Availability

The original contributions presented in the study are included in the article/supplementary material, further inquiries can be directed to the corresponding author/s.
