# Supplementary material for: An FTA card-based CRISPR/Cas12a platform with isothermal amplification for portable tuberculosis diagnosis
Source: Front Microbiol. 2026 Sep 29;17:1958414. doi: 10.3389/fmicb.2026.1958414 (PMC13658155; doi:10.3389/fmicb.2026.1958414)
Supplement: Supplementary file 1 [file Data_Sheet_1.docx]

**An FTA Card-Based CRISPR/Cas12a Platform with Isothermal Amplification for Portable Tuberculosis Diagnosis**

Jiayin Xing^1†^, Xiangxiang Zhao^1^^†^, Gaojie Wang^1^, Shilong Ren^1^, Lei Chen^2^, Wenbin Diao^2^, Meihua Qu^2^, Zhimin Chang^1^, Xiaoqian Wang^1^, Shihao Zhao^1^, Yong Zhang^2*^, Ningning Song^1^^*^

^1^Weifang Key Laboratory of Respiratory Tract Pathogens and Drug Therapy, School of Life Science and Technology, Shandong Second Medical University, Weifang, China

^2^Weifang NO.2 people’s hospital, Weifang, Shandong 261041, P.R. China.

^†^These authors contributed equally to this work.

*** Correspondence:**

Ningning Song, songningning@sdsmu.edu.cn; Yong Zhang, wfeyzy@163.com.

***Supplementary Material***

**Supplementary Table1. PCR primers for preparation of standard templates**

| Name | Sequence (5′→3′) |
| --- | --- |
| IS6110-dsDNA-F | CCGAGACGATCAACGGCCTATACAAGACCGAGCTGATCAAACCCGGC  AAGCCCTGGCGG |
| IS6110-dsDNA-R | CCGCCAGGGCTTGCCGGGTTTGATCAGCTCGGTCTTGTATAGGCCGTTGATCGTCTCGG |
| gyrB-  dsDNA-F | CGAGAACGGGCATGTGTTTTTGGCACAACCGCCGCTGTACAAACTCAA  GTGGCAGCGCA |
| gyrB-  dsDNA-R | TGCGCTGCCACTTGAGTTTGTACAGCGGCGGTTGTGCCAAAAACACATG  CCCGTTCTCG |
| esxB-  dsDNA-F | CGCGCAGGAGGCAGGTAATTTCGAGCGGATCTCCGGCGACCTGAAAAC  CCAGATCGACC |
| esxB-  dsDNA-R | GGTCGATCTGGGTTTTCAGGTCGCCGGAGATCCGCTCGAAATTACCTGC  CTCCTGCGCG |
| ASdes-  T-F | ATAGAGGACGGAGTCGGTGAGGCTCTCCGCGAAATAGTGGCCCTGGTG  GTTTTGGCCTG |
| ASdes-  T-R | AGAAACTTCGCCTCGAGGTAGTCAACCGGGGCTTCAGCCCAGGCCAAA  ACCACCAGGGC |

**Supplementary Figure S1. SDS–PAGE analysis of purified recombinant Cas12a protein (~138 kDa).** Lane M, molecular weight marker; lane Cas12a, purified non-reduced Cas12a protein.

**Supplementary Table2. Comparison of the present FTA–ERA/RT-ERA–CRISPR/Cas12a platform with previously reported CRISPR-based tuberculosis diagnostic methods.**

| **Detection method** | **Target** | **Sample type** | **Extraction requirement** | **Amplification** | **Readout** | **LOD** | **Detection time** | **Ref.** |
| --- | --- | --- | --- | --- | --- | --- | --- | --- |
| ERA-CRISPR/Cas12a | IS6110 | Clinical samples | Required | ERA | Fluorescence/LFA | 9 copies/μL | ~50 min | [15] |
| CRISPR/Cas12a | MTB DNA | Clinical samples | Required | PCR/LAMP | Fluorescence | - | Several hours | [37] |
| CRISPR-based assay | MTB target | Sputum | Extraction-free | Amplification-free | Visual | - | Rapid | [8] |
| FTA–ERA/RT-ERA–CRISPR/Cas12a | IS6110/esxB/gyrB/ASdes | Serum | FTA card-based simplified processing | ERA/RT-ERA | Fluorescence & LFA | 193,97,387,116 copies/μL | ~45–60 min | This study |
